# Microsurgery Competency During Plastic Surgery Residency: An Objective Skills Assessment of an Integrated Residency Training Program

**Published:** 2018-09-25

**Authors:** Matthew A. Applebaum, Erin L. Doren, Ali M. Ghanem, Simon R. Myers, Michael Harrington, David J. Smith

**Affiliations:** ^a^Department of Plastic Surgery, Virginia Tech Carilion, Roanoke; ^b^Department of Plastic Surgery, Medical College of Wisconsin, Milwaukee; ^c^BARTS and The London School of Medicine and Dentistry at the Queen Mary University, London, United Kingdom; ^d^Division of Plastic Surgery, Department of Surgery, University of South Florida, Tampa

**Keywords:** microsurgery training, resident education, microsurgery, hand-motion analysis, education

## Abstract

**Objective:** Microsurgical education is an integral aspect of plastic surgery training. Like most traditional surgical education models, microsurgical skills are taught on an apprenticeship model. This study aims at evaluating microsurgery skill acquisition within an integrated plastic surgery residency using electromagnetic hand-motion analysis and a global rating scale. **Methods:** This is a cross-sectional study of an integrated plastic surgery residency program. Participants performed microsurgical arterial anastomoses on cryopreserved rat aortas. Hand-motion analysis was recorded using a trakSTAR hand-motion tracker. Total time to complete the task, number of hand movements, and path length (mm) were recorded. Participant videos were graded using a subjective global rating scale (scored 0-100). **Results:** The data demonstrated construct validity, as hand-motion analysis outcome measures statistically varied according to the level of skill. Mean global rating scale scores increased with level of experience but lacked statistical significance. **Conclusions:** These data suggest that the objective assessment of hand motion is a valid tool for the evaluation of microsurgical skill. It is more accurate and reflective of the level of skill than a global rating scale. Identifying the predictive validity of hand-motion analysis will be a useful tool to establish clinical safe training and practice thresholds, and the application of both assessment tools simultaneously can yield better evaluation.

Since the introduction of contemporary microsurgery in the 1960s, microsurgical education has been an integral and essential aspect of training in plastic and reconstructive surgery. Many would agree that microsurgery is one of the most complex and technically demanding of all surgical techniques. However, like most traditional surgical education, microsurgical skills are still taught on an apprenticeship model in the operating room.^1^^,^^2^ As patient safety and the ethics of “practicing” on patients have become more apparent, along with the advent of restricted training hours, there is need for surgical curricula to become competency- and potentially simulation-based.

Criteria-based assessments that use checklists and global rating scales (GRSs) are a subjective way to assess microsurgical competency and are often used in microsurgical training courses and plastic surgery curricula.^3^^-^14 Incorporating more objective assessments, such as hand-motion analysis, may be a more effective way to assess improvement in performance and competency of microsurgical skill and give better insight into a learner's technique. Hand-motion analysis tools have been previously studied and validated, to varying degrees, for the assessment of microsurgical proficiency.^4^^,^^15^^-^18 None have yet been implemented to measure core competency in plastic surgery training.

This study aims at evaluating microsurgery skill acquisition within a US integrated plastic surgery residency training program using electromagnetic hand-motion analysis and a validated GRS. We hypothesize that hand-motion analysis and GRS scores will correlate with successful progression in the integrated residency training program. Hand-motion analysis may be integrated with the traditional competency assessment, the GRS, to create a superior evaluation tool.

## METHODS

This is a cross-sectional study from a US integrated 6-year plastic surgery residency program. Participants included all current residents of the program postgraduate years (PGY) 1 to 6 and assistant and associate professor faculty in the department of plastic surgery. The study was conducted over the course of 1 year, 2015. Participants completed a survey about their prior microsurgical experience and demographic data such as age, hand dominance, and level of training. The number of microsurgical cases was recorded from the resident case logs. The number of microsurgical cases was self-reported by faculty. Participants then performed 3 consecutive microsurgical arterial anastomoses on a cryopreserved rat aorta using 8.0 nylon suture and the 180-halving technique. A video of the anastomoses was recorded via standard recording equipment.

**Figure 1:**
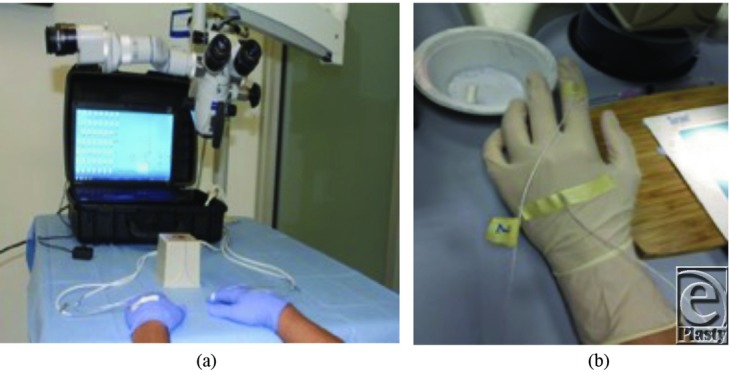
(a) Electromagnetic hand-motion analysis device and setup. (b) Hand electrode placement.

The first anastomosis was considered a warm-up or practice session. Hand-motion analysis of the second and third consecutive anastomoses was averaged and recorded using the trakSTAR 3-dimensional (3D) electromagnetic motion-tracking device (Ascension Technology, Shelburne, Vermont) and analyzed using customized and validated software.^19^^,^^20^ The hand-motion tracker uses an electromagnetic field transmitter and 4 tracking sensors. Two sensors were placed on each participant's dominant and nondominant hands, one on the dorsum of the third metacarpal and another on the middle phalanx of the index finger (Fig 1). Total time to complete the task (minutes), number of hand movements, and path length (millimeters) were recorded by the computer software and were used as outcome measures.

**Figure 2:**
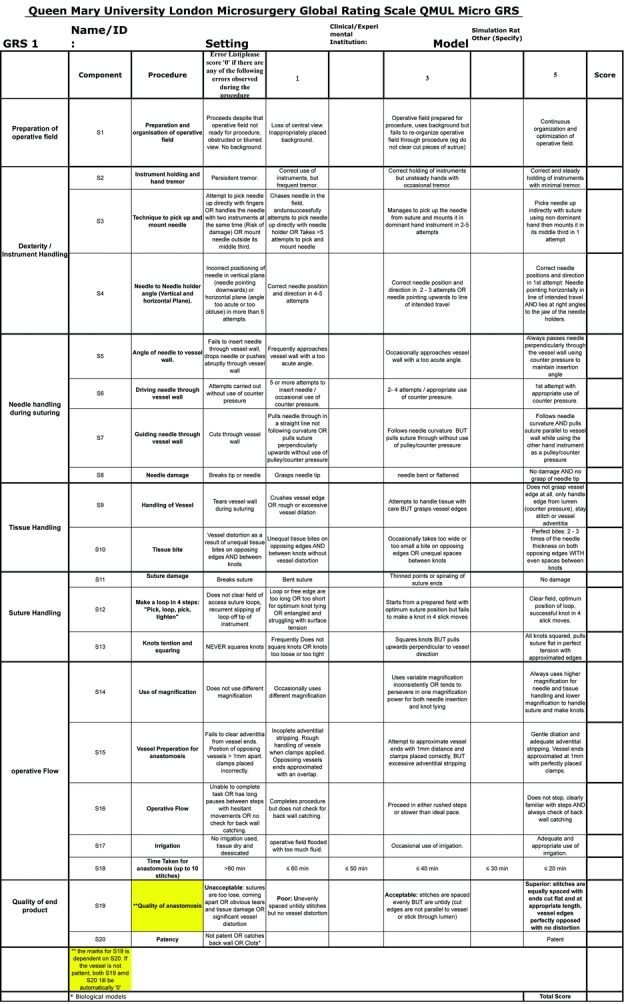
Queen Mary University London microsurgery global rating scale.

The videotapes of the participants’ microsurgical anastomoses (either their second or third anastomosis was randomly selected) were subsequently blinded and reviewed by 3 different experienced raters (plastic surgery faculty and microsurgery laboratory faculty) utilizing the GRS for microsurgery developed by Queen Mary University of London (QMUL)^21^^,^^22^ (Fig 2). This scale includes 20 components assessing preparation of the operative field, dexterity/instrument handling, needle handling, tissue handling, suture handling, operative flow, and quality of end product. The GRS measures each of the 20 outcomes on a 5-point Likert scale, making the total range of scores 0% to 100%. The raters were trained by an experienced faculty member on use of the GRS for evaluation.

Data were analyzed using SPSS (version 21). Analysis of variance and Bonferroni post hoc analysis were used to compare the mean scores between the participant groups. A *P* value of less than .05 was considered significant.

## RESULTS

A total of 18 residents and 4 faculty members participated in this study and were stratified by their current experience level. All completed videotaped microsurgical anastomoses in the laboratory along with hand-motion analysis. All but one participant were right-handed. The mean number of microsurgical cases performed during residency by level of training is as follows: PGY1, n = 0, PGY2, n = 1; PGY3, n = 18; PGY4, n = 3; PGY5, n = 18; and PGY6, n = 44. The mean number of microsurgical cases performed by faculty participants in the last 5 years is 107 (range, 0-250).

**Figure 3:**
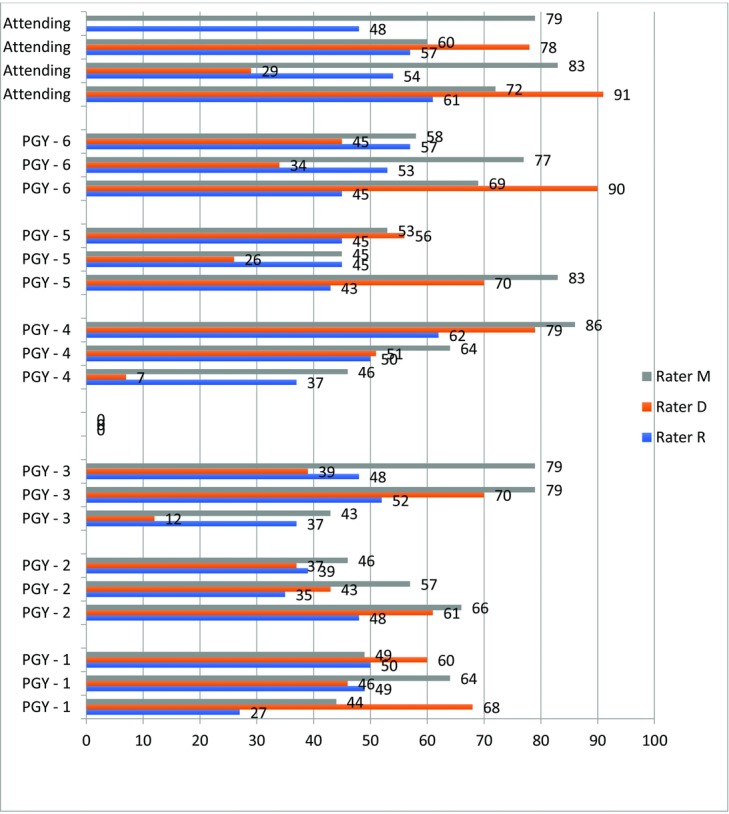
Global rating scale scores stratified by level of training (descending order, attending, PGY6-1). PGY indicates postgraduate year.

The GRS scores for each level of participant by each individual rater are shown in Figure 3. The mean GRS score for entry-level residents (PGY1-3) was 50 (range, 27-79). The mean score for upper-level residents (PGY4-6) was 55 (range, 37-62). The mean score for faculty participants was 65 (range, 46-78). A difference between groups existed, with the mean score increasing with level of experience; however, this lacked statistical significance (*P* = .998).

**Figure 4:**
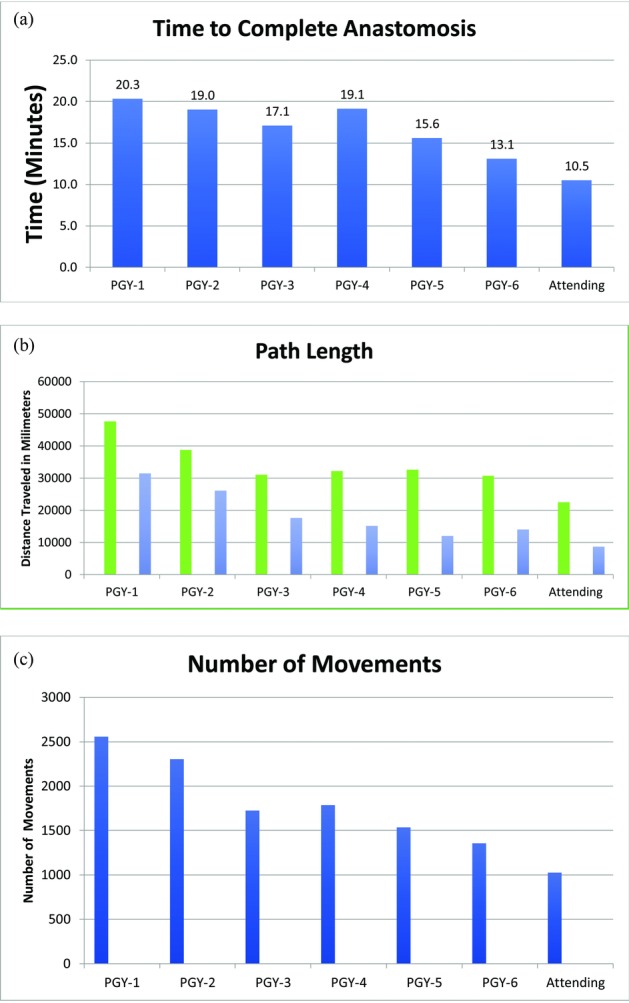
Hand-motion analysis results. (a) Average time to complete anastomosis (minutes). (b) Total path length traveled, dominant hand (green), and nondominant hand (blue). (c) Total number of hand movements. PGY indicates postgraduate year.

Hand-motion analysis data were stratified by level of training. The mean time to complete a single anastomosis (minutes), total number of hand movements, and path length (millimeters) decreased with increased level of training (Fig 4). The difference between the participant groups’ mean time, number of hand movements, and path length was statistically significant (*P* < .001). A post hoc test showed that the time to complete anastomosis was significantly lower in the PGY6 (14.17 ± 2.04 minutes, *P* = .03) and attending groups (17.78 ± 6.11 minutes, *P* < .001) than in the PGY1-3 (23.10 ± 7.02) and PGY4-5 groups (18.13 ± 3.92). This demonstrates construct validity, as hand-motion analysis outcome measures statistically varied according to the level of skill for time, number of hand movements, and path length.

## DISCUSSION

Microsurgical training courses are an integral adjunct to plastic surgery residency training.^6^^,^^18^^,^^23^^-^28 Microsurgery courses have traditionally consisted of subjective measurements in the form of logbooks and clinical observation.^12^ More recently, institutions have incorporated checklists and GRSs in an attempt to improve the objectivity in the assessment of its trainees.^4^^-^11^,^^13^^,^^14^^,^^18^ However, there proves to be limited standardization between training courses, rating scales used, and assessment of proficiency. This study evaluates microsurgery skill acquisition within an integrated plastic surgery residency program using electromagnetic hand-motion analysis combined with a validated GRS. Ultimately, we seek an evaluation tool that can be used for core competency in plastic surgery training.

This GRS is a subjective measurement tool developed at the QMUL to assess microsurgical skill. This scale was developed and has been validated and reliability tested by researchers at the QMUL.^21^^,^^22^ We chose this competency measure for our assessment because of its ease of use and 0- to 100-point scale. The QMUL GRS combines all of the outcome measures used in previously reported scales into 1 checklist.^18^ These measures include preparation of the operative field, dexterity/instrument handling, needle handling, tissue handling, suture handling, operative flow, and quality of end product.^18^ Additional benefits of utilizing the QMUL scale are enabling the assessment of nuances of the anastomosis that hand-motion analysis cannot track, such as angle of the needle to the vessel wall and use of magnification. The scale also tests proficiency by assessing the quality of the end product and patency of the anastomoses. The GRS scores, from blinded assessment, did separate out level of microsurgical skill appropriately by experience level, with the entry-level residents having a mean score of 50, upper-level residents having a mean score of 55, and plastic surgery faculty having a mean score of 65. Yet, this did not reach statistical significance. This is most likely explained by small sample size and poor interrater reliability, as scores for individual participants varied greatly depending on the rater.

As microsurgical skills consist of fine motor movements, we took our study a step further to look at the efficiency of hand motion using the trakSTAR 3D electromagnetic motion-tracking device (Ascension Technology). The tracker measures dexterity data including economy of hand motion and hand travel distance. Previous studies support the use of hand-motion analysis to assess microsurgical skill.^4^^,^^5^^,^^7^^,^^15^^,^^16^^,^^19^^,^^20^^,^^28^ This stems from the notion that technical performance is bettered through the use of operant conditioning.^28^ To prove the applicability of hand-motion analysis as a training tool, Grober et al^17^ were able to show that objectively measured skills via hand-motion analysis within the laboratory were measured with equal results within the operating room.

The objective data collected from the hand-motion analysis in addition to the GRS were hypothesized to be strong predictors in analyzing the experience level of trainees in microsurgery. Previous literature supports a correlation between the GRS and hand-motion analysis scores.^10^^-^12^,^^15^^,^^16^ Although our study did not find a statistically significant difference between groups using the GRS, we did find that groups differed significantly in regard to hand-motion analysis. In our plastic surgery curriculum, involvement with microsurgical cases really begins during the third year, with PGY6 residents frequently performing microsurgery procedures autonomously, which likely contributed to this finding. These data suggest that hand-motion analysis can accurately measure microsurgery skill, as economy of hand motion and anastomoses time improved with year of surgical training (Fig 4).

Hand-motion analysis may help overcome the limitations of assessment with a GRS alone, such as an inconsistency between raters and time-consuming assessments. However, this technology is not without its limitations. Although this technology is able to efficiently and correctly collect data to analyze the experience one has in microsurgery, it is costly.^17^^,^^18^ The inability to assess patency has also limited its widespread use in training programs. McGoldrick et al^28^ proposed the use of computer software that measures dexterity and economy of hand motion without the use of hand sensors. They argued that tracking the needle driver tip, while making an anastomosis, was more accurate than the use of hand-motion analysis, believing that outside factors may interfere with the measurement of efficiency when using the hand-motion analysis compared with tracking the needle driver tip.^28^ Other cheap assessment tools for the “end product” have been validated in a similar nonliving model.^26^ With the move toward the reduction, replacement, and refinement of the use of animals in surgical training, it would be necessary to evaluate the cost-effectiveness of these several assessment tools and their predictive validity.^18^

Predictive validity is an important aspect of skills assessment tools. An important limitation of the GRS and hand-motion analysis scores is the need to correlate them to physiological and clinical outcomes. Microsurgery involves a bundle of skills beyond microvascular anastomosis. In this study, the standardized task assessed was only an end-to-end microvascular anastomosis on a cryopreserved animal model. It will be important to correlate semiobjective and objective assessment tools to clinical outcome. Our study used the clinical exposure and assumed competency acquired during an integrated residency program to test for concurrent validity of these assessment tools. Despite the costs, technical challenges of hand-motion measurement, lack of conclusive predictive validity, and inconsistencies with the GRS graders measurement, this study presents an evidence-based appraisal of an entire residency program in relation to microvascular anastomosis skills. Future studies evaluating a larger number of residents in a prospective fashion along the continuum of their training will be needed to further test the utility of this educational tool.

## CONCLUSION

These data suggest that the objective assessment of hand motion is a valid tool for the evaluation of microsurgical skill. It is more accurate and reflective of the level of skill than a GRS. Identifying the predictive validity of hand-motion analysis will be a useful tool to establish clinical safe training and practice thresholds, and the application of both assessment tools simultaneously can yield better evaluation.
